# Supplementary material for: Diverse but unique astrocytic phenotypes during embryonic stem cell differentiation, culturing and development
Source: Commun Biol. 2023 Jan 13;6:40. doi: 10.1038/s42003-023-04410-3 (PMC9839673; doi:10.1038/s42003-023-04410-3)
Supplement: Supplementary file 2 — Supplementary Information [file 42003_2023_4410_MOESM2_ESM.pdf]

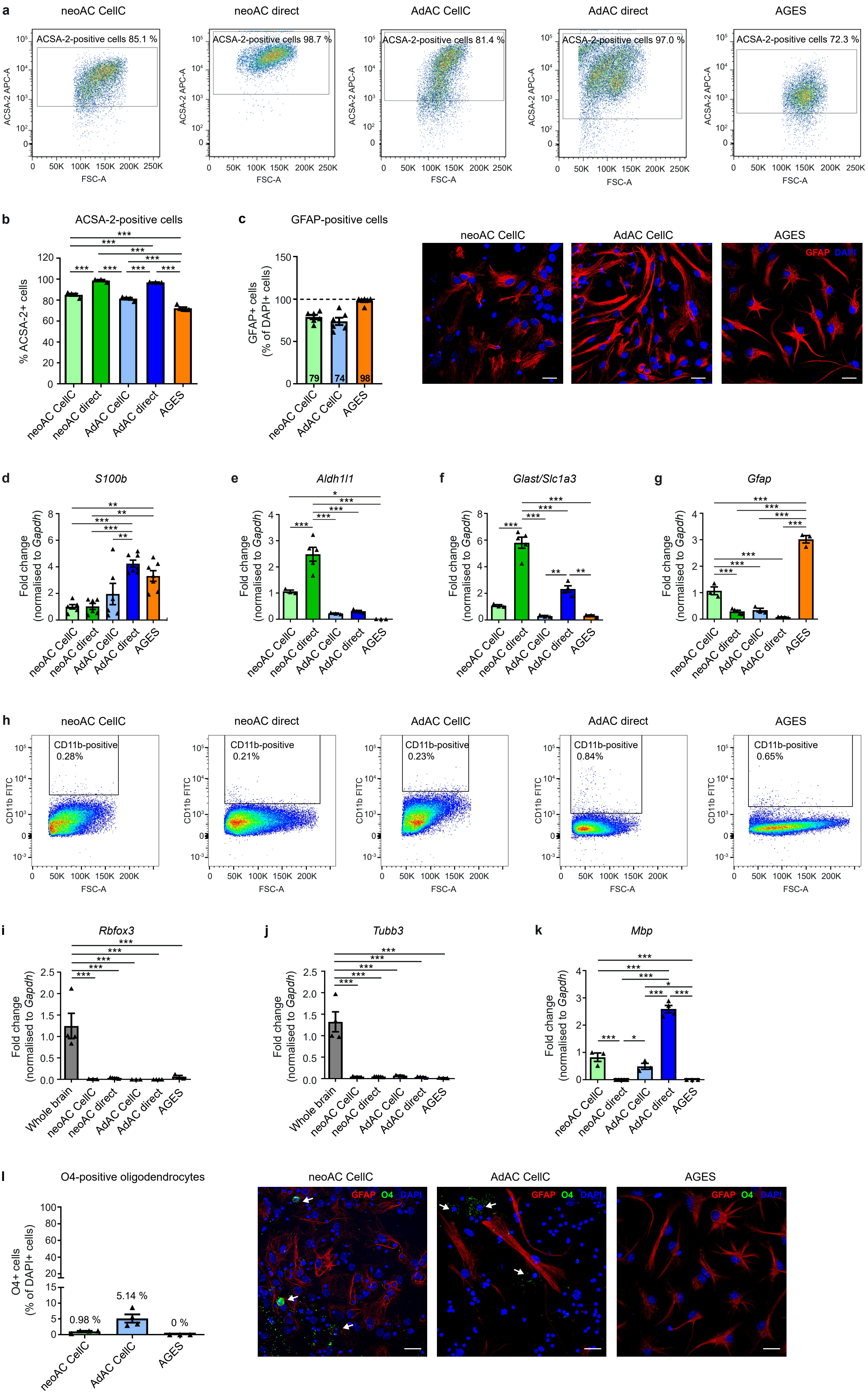

a
neoAC CellC
ACSA-2-positive cells 85.1 %
neoAC direct
ACSA-2-positive cells 98.7 %
AdAC CellC
ACSA-2-positive cells 81.4 %
AdAC direct
ACSA-2-positive cells 97.0 %
AGES
ACSA-2-positive cells 72.3 %
ACSA-2 APC-A
FSC-A
b
ACSA-2-positive cells
% ACSA-2+ cells
c
GFAP-positive cells
GFAP+ cells (% of DAPI+ cells)
79
74
98
GFAP DAPI
d
S100b
e
Aldh1l1
f
Glast/Slc1a3
g
Gfap
Fold change (normalised to Gapdh)
neoAC CellC
neoAC direct
AdAC CellC
AdAC direct
AGES
h
CD11b-positive 0.28%
CD11b-positive 0.21%
CD11b-positive 0.23%
CD11b-positive 0.84%
CD11b-positive 0.65%
CD11b FITC
i
Rbfox3
j
Tubb3
k
Mbp
Whole brain
l
O4-positive oligodendrocytes
O4+ cells (% of DAPI+ cells)
0.98 %
5.14 %
0 %
GFAP O4 DAPI

Supplementary Figure 1: Cell purity and cell type composition changes during cell culturing, development and cell differentiation.

a Fluorescence activated cell sorting (FACS) of ACSA-2 labelled (stained) cell suspensions is shown in cultured neonatal astrocytes (neoAC CellC), directly isolated neonatal astrocytes (neoAC direct), cultured adult astrocytes (AdAC CellC), directly isolated adult astrocytes (AdAC direct) and AGES. Representative FACS blots are shown for each cell type which include the average percentage of ACSA-2-positive cells. **b** Quantities of ACSA-2-positive cells in primary astrocytes and AGES as determined by FACS were summarized. neoAC CellC (n=4), neoAC direct (n=3), AdAC CellC (n=4), AdAC direct (n=3), AGES (n=5); ****P* < 0.001. **c** Cultured neonatal (neoAC CellC) and adult (AdAC CellC) astrocytes and AGES were immunostained for GFAP (red) and the cell nuclei marker DAPI (blue). The number of GFAP-positive cells as the percentage of all DAPI-positive cells was determined by Fiji analysis. The average percentage of GFAP-positive cells is displayed. Representative images are shown. Scale bar = 25 µm; neoAC CellC (n=6), AdAC CellC (n=6), AGES (n=6). **d-g** Gene expression of the astrocyte markers **d** ***S100b*** (AGES vs. neoAC CellC ***P* = 0.0051, AGES vs. neoAC direct ***P* = 0.0056, AdAC CellC vs. AdAC direct ***P* = 0.0057, ****P* < 0.001), **e** *Aldh1l1* (**P* = 0.0130; ****P* < 0.001), **f** *Slc1a3/Glast* (AdAC CellC vs. AdAC direct ***P* = 0.0034; AdAC direct vs. AGES ***P* = 0.0037; ****P* < 0.001) and **g** *Gfap* (****P* < 0.001) was determined by quantitative real-time PCR. All expression values were normalized to the internal control *Gapdh* and the fold change compared to cultured neonatal astrocytes (neoAC CellC) was displayed. neoAC CellC (n=3-6), neoAC direct (n=5-6), AdAC CellC (n=3-6), AdAC direct (n=4-7), AGES (n=3-7). **h** Representative FACS blots of CD11b labelled cell suspensions in neoAC CellC, neoAC direct, AdAC CellC, AdAC direct and AGES are shown for each cell type which include the average percentage of ACSA-2-positive cells; neoAC CellC (n=4), neoAC direct (n=5), AdAC CellC (n=6), AdAC direct (n=6), AGES (n=3). **i-j** Gene expression of the neuronal markers **i** *Rbfox3* (NeuN) and **j** Tubb3 was determined by quantitative real-time PCR. All expression values were normalized to the internal control *Gapdh* and whole brain lysates as a reference. Whole brain (n=4), neoAC CellC (n=3), neoAC direct (n=5), AdAC CellC (n=3), AdAC direct (n=4), AGES (n=3); ****P* < 0.001. **k** *Mbp* was used as an oligodendrocytic marker. All expression values were normalized to the internal control *Gapdh* and neoAC CellC. neoAC CellC (n=3), neoAC direct (n=5), AdAC CellC (n=3), AdAC direct (n=4), AGES (n=3); neoAC direct vs. AdAC CellC **P* = 0.0179, AGES vs. AdAC CellC **P* = 0.0352, ****P* < 0.001. **l** Cultured neonatal (neoAC CellC) and adult (AdAC CellC) astrocytes and AGES were immunostained for the oligodendrocyte marker O4 (green), GFAP (red) and the cell nuclei marker DAPI (blue). The number of O4-positive cells as the percentage of all DAPI-positive cells was determined by Fiji analysis. The average percentage of O4-positive cells is displayed. Representative images are shown. Scale bar = 25 µm. Mean ± SEM; neoAC CellC (n=4), AdAC CellC (n=4), AGES (n=5). Mean ± SEM, ANOVA with Tukey´s post hoc test.

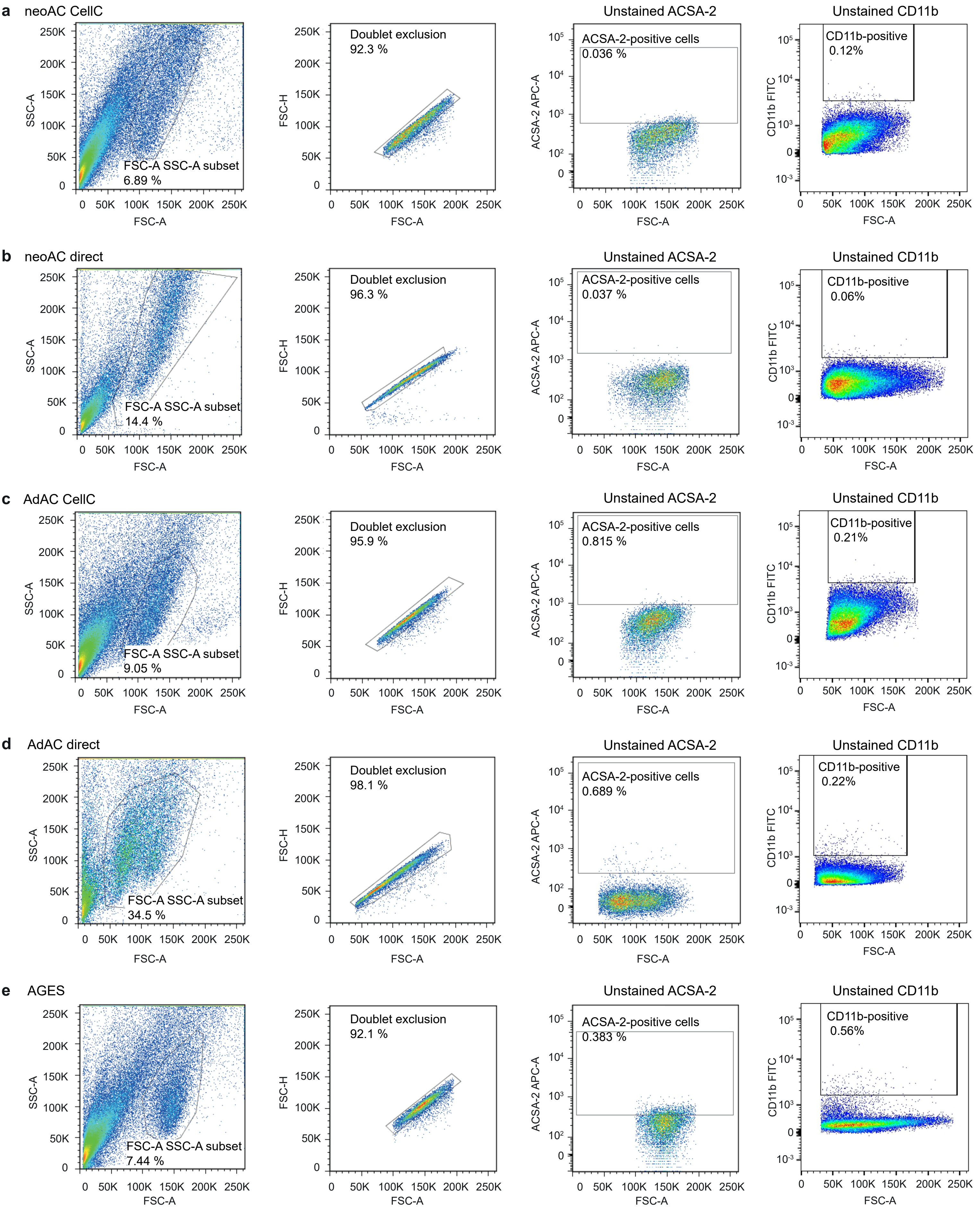

Supplementary Figure 2: Gating strategy.

a-e The gating strategy for fluorescence activated cell sorting (FACS) of ACSA-2 or CD11b-labelled (stained) and unlabelled (unstained) cell suspensions is shown, demonstrating doublet exclusion (2. graph) and gating for APC-labelled ACSA-2-positive cells (Supplementary Fig. 1a, d) based on ACSA-2 negative stain (3. graph) or CD11b negative stain (4. graph) in **a** cultured neonatal astrocytes (neoAC CellC), **b** directly isolated neonatal astrocytes (neoAC direct), **c** cultured adult astrocytes (AdAC CellC), **d** directly isolated adult astrocytes (AdAC direct) and **e** AGES. Representative FACS blots are shown for each cell type which include the average percentage of ACSA-2 or CD11b-positive cells.

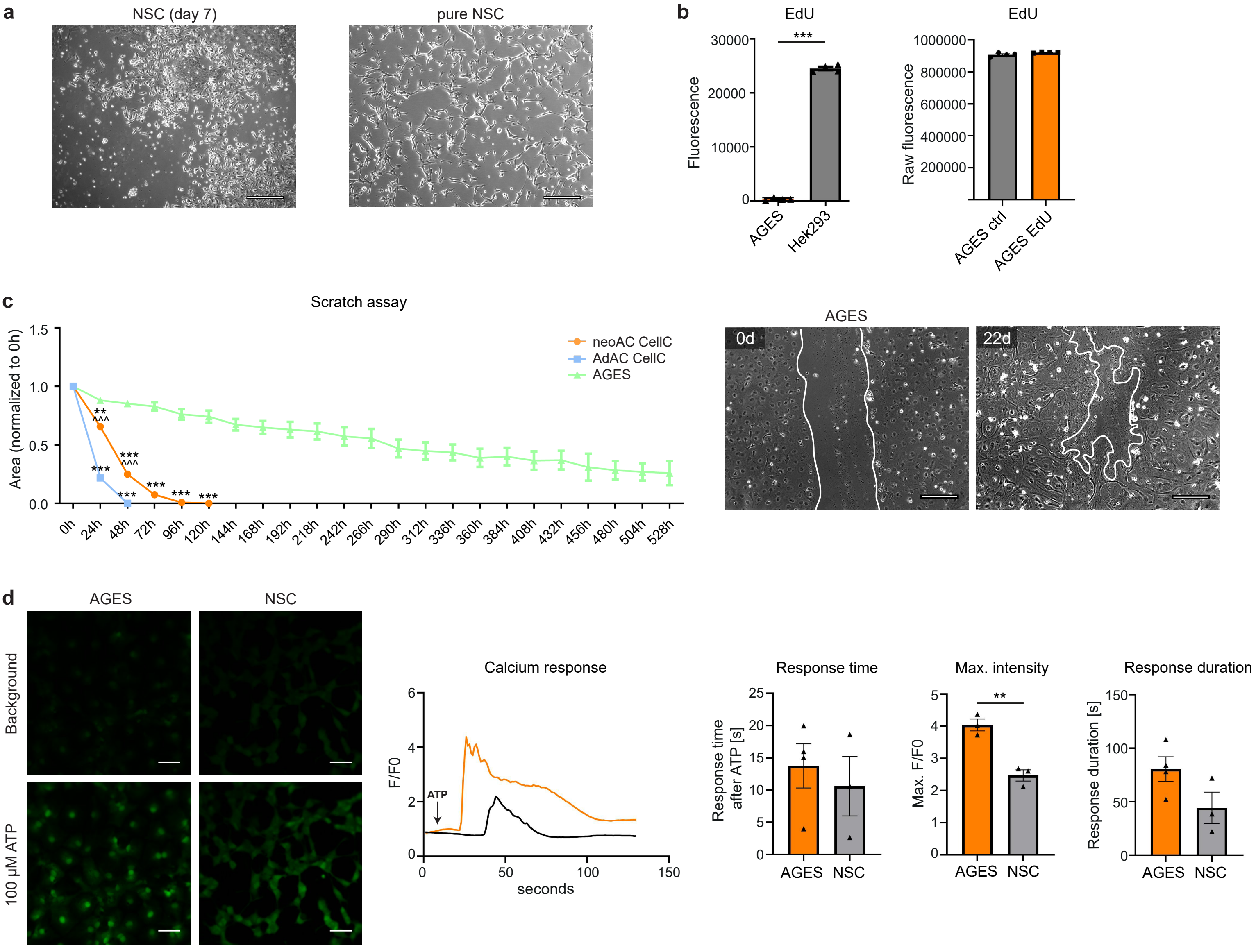

Supplementary Figure 3: Migratory behavior of AGES and calcium response of NSCs.

a NSCs were differentiated from mESCs. For receiving pure bipolar NSC cultures, NSCs were cultured for at least eight passages in medium containing the growth factors EGF and FGF. Representative phase contrast images of NSC 7 days after starting the differentiation (mixed mESC and NSC culture) and pure NSC cultures are shown. Scale bar = 200 µm. **b** Proliferation was measured by fluorescent immunolabeling for the thymidine analogue EdU. Fluorescent signal was normalized to background absorbance. Fluorescence signal without normalization (raw fluorescence) is shown to compare the EdU with the no EdU (ctrl) condition. Two-tailed t-test,****P* < 0.001. **c** A scratch assay was used to determine the migratory behaviour of cultured neonatal astrocytes (neoAC CellC), cultured adult astrocytes (AdAC CellC) and AGES. The area of the wound gap normalized to time point 0 h is shown over time. * significance compared to AGES; ^ significance compared to AdAC CellC. Mean ± SEM; neoAC CellC (n=4), AdAC CellC (n=4), AGES (n=4); Two-way ANOVA with Bonferroni post hoc test; ***P* = 0.004; ****P* < 0.001; ^^^*P* < 0.001. Representative phase contrast images of the wound gap at the starting point (0 d) and after 22 days are shown of AGES. Scale bar = 200 µm. **d** Calcium signaling of AGES and NSC was determined by calcium imaging using Fluo-4, AM as a calcium indicator and 100 µM ATP as a stimulus for calcium release. Representative images are shown before ATP stimulation (= background) and after ATP stimulation. Scale bar = 50 µm. The fluorescence intensity normalized to background fluorescence (F/F0) is shown over time for each cell type. The time until cells responded with a calcium peak was measured (*P* > 0.05). The maximum fluorescence intensity was compared between all cell types (***P* = 0.0035). The time until cells returned to baseline levels was determined (*P* > 0.05). Mean ± SEM, ANOVA with Tukey´s post hoc test; AGES (n=4), NSC (n=3).

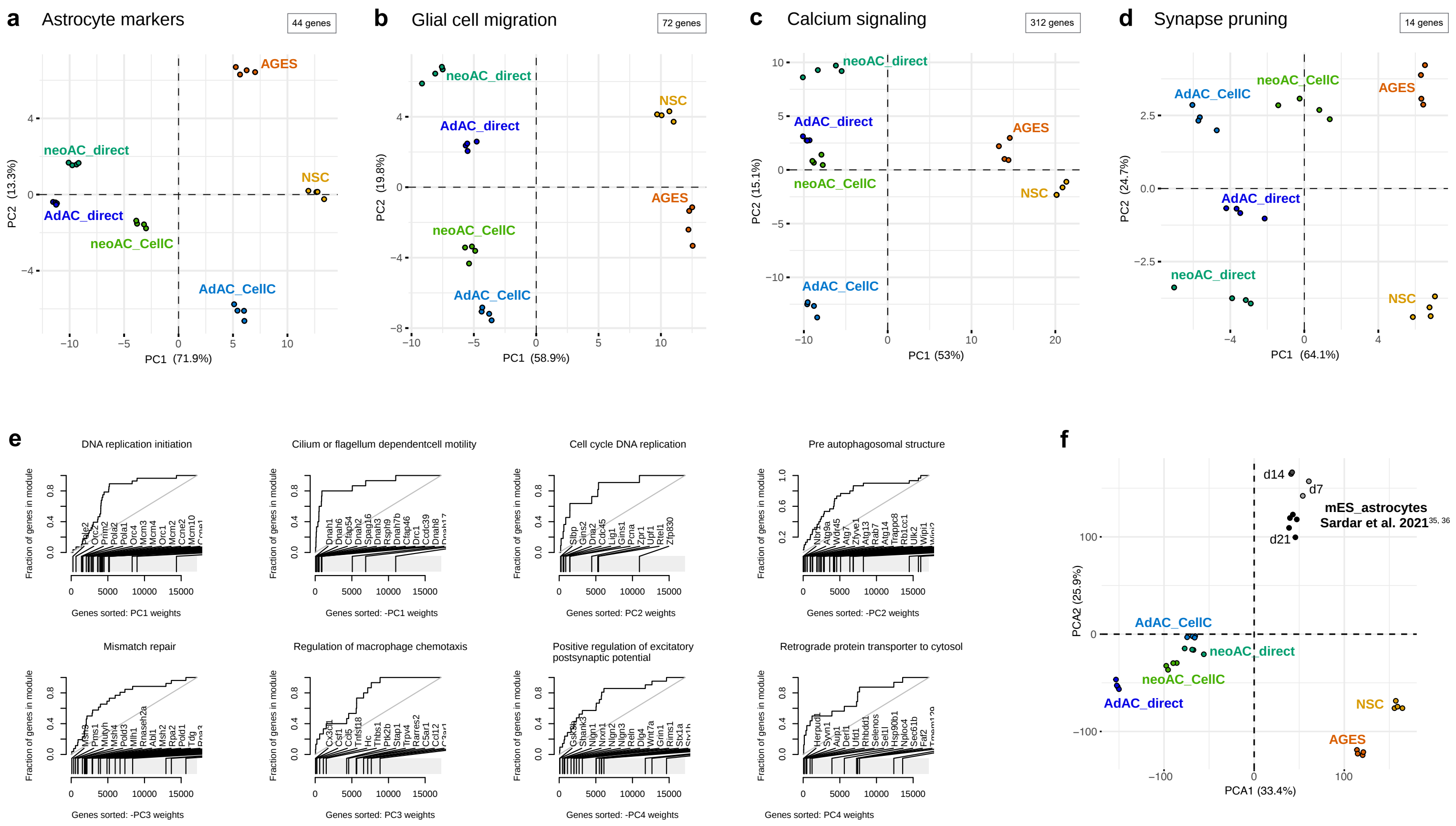

Supplementary Figure 4: Principal component analysis and evidence plots of RNA-seq data underline functional differences between astrocyte cell types.

RNA-seq was performed from cultured neonatal astrocytes (neoAC CellC; n=4), directly isolated neonatal astrocytes (neoAC direct; n=4), cultured adult astrocytes (AdAC CellC; n=4), directly isolated adult astrocytes (AdAC direct; n=4), AGES (n=4) and NSCs (n=4). **a** Principal component analysis (PCA) of published astrocyte marker genes [11] is shown. **b-d** Principal component analysis (PCA) of samples based only on genes from select GO terms relating to our functional assays was performed. The variance explained by the first and second component is shown on the x- and y-axis. **e** Evidence plots of select GO terms from Fig. 3d,e displaying genes sorted based on the gene PCA loadings on the x-axis and cumulative fraction of genes from a particular gene set (GO term) on the y-axis. **f** PCA of our samples and mouse embryonic stem cell-derived astrocytes (mES_astrocytes) from different time points of culture (day 7, 14, 21) published by Sardar et al. [35, 36].

	neoAC CellC	neoAC direct	AdAC CellC	AdAC direct	AGES	NSC	Vellis neoAC
**Astrocytic marker profile**							
GFAP protein levels	↑	↑	↑	↑	↑↑↑		
S100B protein levels	↑	↑	↑	↑↑↑	↑		
GLT-1 protein levels	+/-	+/-	+/-	+/-	+/-		
Gfap gene expression	↑↑	↑	↑	↑	↑↑↑		
Aldh1l1 gene expression	↑↑	↑↑↑	↑	↑	-		
Glast/Slc1a3 gene expression	↑↑	↑↑↑	↑	↑↑	↑		
S100b gene expression	↑	↑	↑	↑↑↑	↑↑↑		
ACSA-2-positive cells	85.1 %	98.7 %	81.4 %	97.0 %	72.3 %		
GFAP-positive cells	79 %		74 %		98 %		
**Astrocyte reactivity**							
Reactivity marker expression	↑	↑	↑	↑	↑		↑↑↑
MCM-induced reactivity	↑↑		↑↑		↑↑↑		
**Non-astrocytic markers**							
CD11b-positive microglia	-	-	-	-	-		
O4-positive oligodendrocytes	0.98 %		5.14 %		-		
Mbp gene expression	↑	-	↑	↑↑	-		
Rbfox3 gene expression	-	-	-	-	-		
Tubb3 gene expression	-	-	-	-	-		
**Functional assays**							
Proliferation	↑↑		↑		↑		
Cell death	+/-		+/-		+/-		
Wound healing	↑		↑↑		↓		
Glucose uptake	+/-		+/-		+/-		
Lactate release	+/-		+/-		+/-		
Synaptosome uptake	+/-		+/-		+/-		
Calcium release response time	↑↑		↑		↑	↑	
Calcium released	↑		↑		↑↑	↑	
Calcium signalling response duration	+/-		+/-		+/-	+/-	
**Transcriptomic changes**							
Microtubule genes	↑↑↑	↑↑	↑↑	↑↑↑	↑	-	
Cell cycle genes	↑↑↑	↑↑	↑↑↑	↑	↑	↑↑↑	
Autophagy genes	-	-	-	-	↑↑↑	↑	
Synaptic vesicle exocytosis regulation genes	↑	↑↑↑	↑	↑↑↑	↑↑↑	-	
Chemotaxis genes	↑↑↑	↑	↑↑↑	↑	↑↑	↑↑	
Mitochondrial respiration genes	↑↑↑	↑↑↑	↑	↑	-	-	
Cell adhesion genes	↑↑↑	↑↑↑	↑	↑	-	-	
Cilium function genes	↑↑↑	↑	↑	↑↑↑	↑	-	
% ciliated cells (staining)	↑↑		↑↑↑		↑		
Cilia length (staining)	↑↑↑		↑		↑		

Supplementary Figure 5: Summary of experimental results comparing cultured and directly isolated primary cells and AGES.

Summary of the comparison of neonatal astrocytes (neoAC CellC), directly isolated neonatal astrocytes (neoAC direct), cultured adult astrocytes (AdAC CellC), directly isolated adult astrocytes (AdAC direct), AGES, NSC and Vellis neonatal astrocytes (Vellis neoAC). The following symbols were used for visualizing the obtained results: Grey box = The assay was not performed for these cells, ↑ low expression/ functional activity, ↑↑ intermediate expression/ functional activity, ↑↑↑ high expression/ functional activity, - no expression, +/- no change in all analyzed groups.

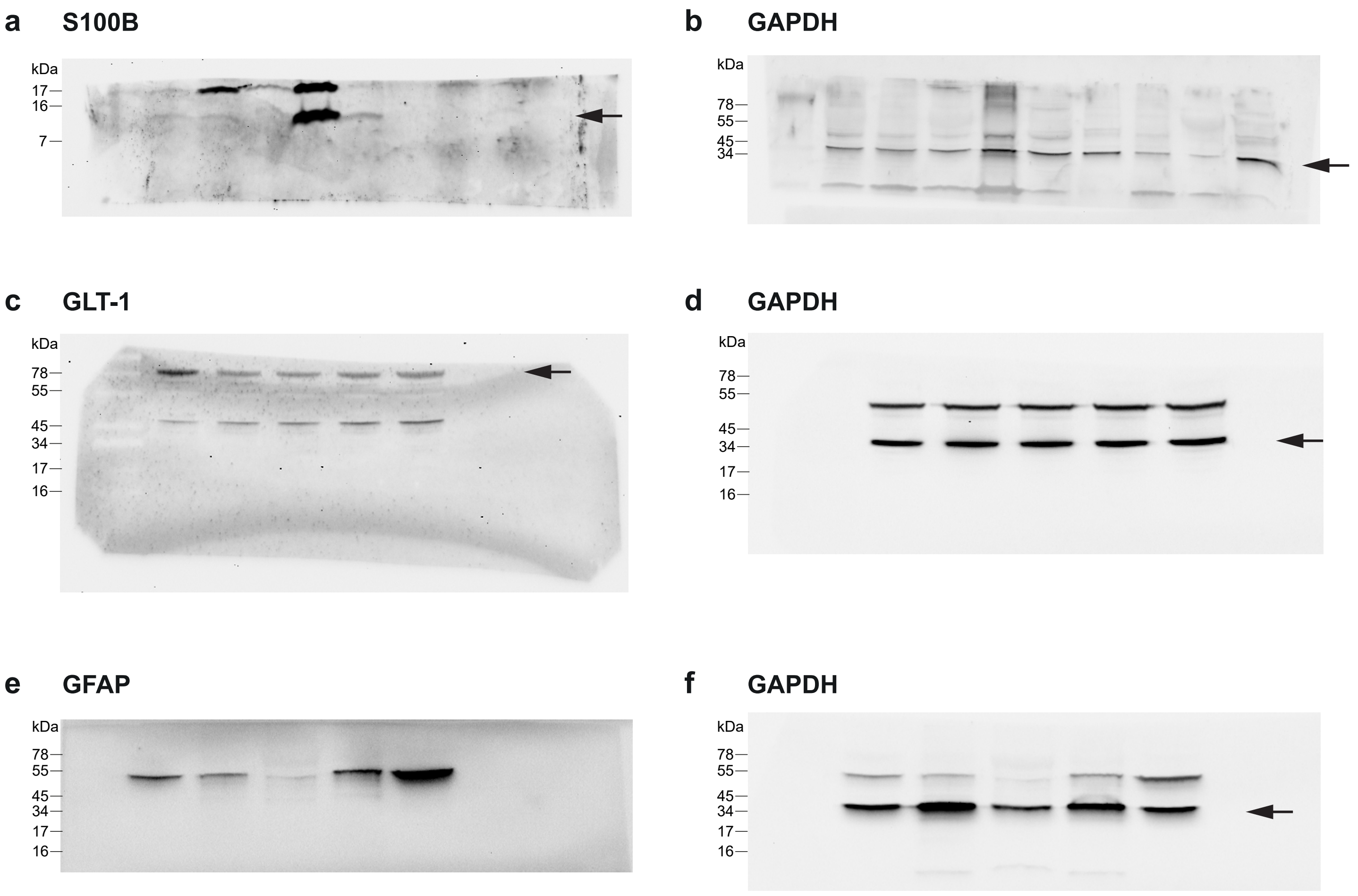

Supplementary Figure 6: Uncropped western blots.

Uncropped western blots of Fig. 1d for S100B are displayed in **a** and the respective GAPDH in **b**. Western blots of Fig. 1e for GLT-1 in **c** and for the respective GAPDH in **d**. Western blots of Fig. 1f are displayed for GFAP in **e** and GAPDH in **f**. The arrow indicates the quantified band.
