# Supplementary material for: Diverse but unique astrocytic phenotypes during embryonic stem cell differentiation, culturing and development
Source: Commun Biol. 2023 Jan 13;6:40. doi: 10.1038/s42003-023-04410-3 (PMC9839673; doi:10.1038/s42003-023-04410-3)
Supplement: Supplementary file 3 — Description of Additional Supplementary Files [file 42003_2023_4410_MOESM3_ESM.docx]

**Description of Additional Supplementary Files**

**File name:** Supplementary Data 1

**Description:** Astrocyte gene list used in Supplementary Figure 4a

**File name:** Supplementary Data 2

**Description:** Source Data for Figure 3d,e

**File name:** Supplementary Data 3

**Description:** Source Data for Figure 3f

**File name:** Supplementary Data 4

**Description:** Source data for Figure 1, 2, 4

**File name:** Supplementary Data 5

**Description:** Source data for Figure 3f: Differential gene expression
