# Supplementary material for: Diverse but unique astrocytic phenotypes during embryonic stem cell differentiation, culturing and development
Source: Commun Biol. 2023 Jan 13;6:40. doi: 10.1038/s42003-023-04410-3 (PMC9839673; doi:10.1038/s42003-023-04410-3)
Supplement: Supplementary file 9 — Reporting summary [file 42003_2023_4410_MOESM9_ESM.pdf]

nature portfolio

Corresponding author(s): Frank Heppner

Last updated by author(s): Oct 24, 2022

Reporting Summary

Nature Portfolio wishes to improve the reproducibility of the work that we publish. This form provides structure for consistency and transparency in reporting. For further information on Nature Portfolio policies, see our Editorial Policies and the Editorial Policy Checklist.

Statistics

For all statistical analyses, confirm that the following items are present in the figure legend, table legend, main text, or Methods section.

n/a	Confirmed	
☐	☒	The exact sample size (n) for each experimental group/condition, given as a discrete number and unit of measurement
☐	☒	A statement on whether measurements were taken from distinct samples or whether the same sample was measured repeatedly
☐	☒	The statistical test(s) used AND whether they are one- or two-sided<br>*Only common tests should be described solely by name; describe more complex techniques in the Methods section.*
☐	☒	A description of all covariates tested
☐	☒	A description of any assumptions or corrections, such as tests of normality and adjustment for multiple comparisons
☐	☒	A full description of the statistical parameters including central tendency (e.g. means) or other basic estimates (e.g. regression coefficient) AND variation (e.g. standard deviation) or associated estimates of uncertainty (e.g. confidence intervals)
☐	☒	For null hypothesis testing, the test statistic (e.g. F, t, r) with confidence intervals, effect sizes, degrees of freedom and P value noted<br>*Give P values as exact values whenever suitable.*
☒	☐	For Bayesian analysis, information on the choice of priors and Markov chain Monte Carlo settings
☐	☒	For hierarchical and complex designs, identification of the appropriate level for tests and full reporting of outcomes
☒	☐	Estimates of effect sizes (e.g. Cohen's d, Pearson's r), indicating how they were calculated

Our web collection on statistics for biologists contains articles on many of the points above.

Software and code

Policy information about availability of computer code

Data collection	BD DIVA software, Infinite i-control Tecan software, QuantStudio™ 6 Flex Real-Time PCR System, TapeStation system
Data analysis	Excel 2016, GraphPad PRISM 5.0, ImageJ, STAR (version 2.7.3a), rlog (DESeq2, version1.22.1), CERNO algorithm from R tmod (0.46.2) package, MsigDB (r msigdbr 6.2.1)

For manuscripts utilizing custom algorithms or software that are central to the research but not yet described in published literature, software must be made available to editors and reviewers. We strongly encourage code deposition in a community repository (e.g. GitHub). See the Nature Portfolio guidelines for submitting code & software for further information.

Data

Policy information about availability of data

All manuscripts must include a data availability statement. This statement should provide the following information, where applicable:

- Accession codes, unique identifiers, or web links for publicly available datasets
- A description of any restrictions on data availability
- For clinical datasets or third party data, please ensure that the statement adheres to our policy

The datasets generated during and/or analysed during the current study are available in the GEO repository, GSE189745.

Field-specific reporting

Please select the one below that is the best fit for your research. If you are not sure, read the appropriate sections before making your selection.

☒ Life sciences ☐ Behavioural & social sciences ☐ Ecological, evolutionary & environmental sciences

For a reference copy of the document with all sections, see nature.com/documents/nr-reporting-summary-flat.pdf

Life sciences study design

All studies must disclose on these points even when the disclosure is negative.

Sample size	Data were generated as multiple exploratory analyses to generate hypotheses and biostatistical planning for future confirmatory studies. Data analysis, processing, descriptive and formal statistical testing were done according to the current customary practice of data handling using Excel 2016, GraphPad PRISM 5.0 and ImageJ.
Data exclusions	Outliers were not excluded.
Replication	Technical replicates were used and several (at least 3) independent experiments were performed.
Randomization	Not relevant as mice were not separated in groups.
Blinding	Investigators were blinded whenever blinding was possible.

Reporting for specific materials, systems and methods

We require information from authors about some types of materials, experimental systems and methods used in many studies. Here, indicate whether each material, system or method listed is relevant to your study. If you are not sure if a list item applies to your research, read the appropriate section before selecting a response.

Materials & experimental systems

n/a	Involved in the study	
☐	☒	Antibodies
☐	☒	Eukaryotic cell lines
☒	☐	Palaeontology and archaeology
☐	☒	Animals and other organisms
☒	☐	Human research participants
☒	☐	Clinical data
☒	☐	Dual use research of concern

Methods

n/a	Involved in the study	
☒	☐	ChIP-seq
☐	☒	Flow cytometry
☒	☐	MRI-based neuroimaging

Antibodies

Antibodies used	GLT-1 (Cell Signaling, 3838S, 1:1000), S100b (Abcam, ab41548, 1:500), GFAP (Abcam, ab4674, 1:1000), O4 (Miltenyi Biotec, 130-115-810, 1:100), ARL13B (Proteintech, 17711-1-AP, 1:1000), gamma-Tubulin (Sigma, T6557, 1:800), ACSA-2 antibody (1:10, Miltenyi Biotec, 130-102-315), FITC-labelled CD11b antibody (1:200, Biolegend, 101206)
Validation	2nd antibody only stainings were performed for each antibody

Eukaryotic cell lines

Policy information about cell lines

Cell line source(s)	Embryonic stem cells derived from C57Bl/6N mice (mESCs; GSC-5003, MTI-GlobalStem)
Authentication	Cell line was bought and not additionally authenticated.
Mycoplasma contamination	Cell lines were negative for mycoplasma contamination.
Commonly misidentified lines (See ICLAC register)	Cell line was not found in the register of misidentified cell lines.

Animals and other organisms

Policy information about studies involving animals; ARRIVE guidelines recommended for reporting animal research

Laboratory animals	C57BL/6J mice, both genders, AdAC: 100-140d or neoAC: p4-8
Wild animals	Wild animals were not included in the study.
Field-collected samples	Mice were group housed under specific pathogen-free conditions on a 12 h light/dark cycle, and food and water were provided to the mice ad libidum.
Ethics oversight	All animal experiments were performed in accordance with the national animal protection guidelines approved by the regional offices for health and social services in Berlin (LaGeSo, license numbers T 0276/07 and O298/17).

Note that full information on the approval of the study protocol must also be provided in the manuscript.

Flow Cytometry

Plots

Confirm that:

☒ The axis labels state the marker and fluorochrome used (e.g. CD4-FITC).

☒ The axis scales are clearly visible. Include numbers along axes only for bottom left plot of group (a 'group' is an analysis of identical markers).

☒ All plots are contour plots with outliers or pseudocolor plots.

☒ A numerical value for number of cells or percentage (with statistics) is provided.

Methodology

Sample preparation	Murine astrocytes were isolated by MACS or differentiated from neural stem cells. For Fluorescence-Activated Cell Sorting (FACS) of cultured cells, cells were detached in PBS using a cell scraper. For FACS analysis directly after cell isolation, cells were collected after flushing from MS columns.
Instrument	BD FACS Canto 2
Software	BD DIVA + FlowJo 7.6.5 software
Cell population abundance	Purity analysis using ACSA-2 and CD11b as markers
Gating strategy	Doublets were excluded by gating before analysing percentage of ACSA-2-positive/ CD11b-positive cells using unstained as negative controls.

☒ Tick this box to confirm that a figure exemplifying the gating strategy is provided in the Supplementary Information.
